# Plastic Surgery Residency Program Instagram Engagement with Prospective Applicants: A Cohort Analysis in the United States

**DOI:** 10.1055/a-2731-4609

**Published:** 2026-01-30

**Authors:** Michael I. Kim, Janine S. Chan, Carly Askinas, Joseph N. Carey, David A. Daar, Emma C. Koesters

**Affiliations:** 1 Keck School of Medicine, University of Southern California, Los Angeles, California, United States; 2 Division of Plastic and Reconstructive Surgery, Keck School of Medicine of University of Southern California, Los Angeles, California, United States

**Keywords:** plastic surgery residency, Instagram, residency recruitment, program reputation

## Abstract

**Background:**

Social media platforms, particularly Instagram, have revolutionized residency recruitment by offering integrated plastic surgery programs innovative avenues to showcase their culture and engage directly with prospective applicants. The COVID-19 pandemic accelerated this digital shift, making virtual interactions essential for evaluating program fit.

**Methods:**

We identified 88 Accreditation Council for Graduate Medical Education-accredited integrated plastic surgery programs for the academic year 2023 to 2024. Instagram posts from January 1, 2020, to October 26, 2024, were reviewed, focusing on “engagement posts” (e.g., meet-and-greets, question-and-answer [Q&A] sessions, open houses, workshops, conference meet-ups). Programs were stratified into cohorts based on Doximity rankings (Top 20, 21–40, 41–60, and 61–88), geographic region, and program size.

**Results:**

All programs maintained active Instagram profiles, collectively sharing 13,719 posts and averaging 32.4 posts per program annually. Among these, 369 (2.7%) were engagement posts, with meet-and-greets accounting for 71.3% of such content. Notably, Top 20 programs posted significantly more frequently (52.6 posts/year) than lower-ranked cohorts (21–40: 35.0, 41–60: 26.8, 61–88: 20.1; *p* < 0.001) and had a higher engagement post rate (3.9% vs. 2.0–2.7%; *p* = 0.033).

**Conclusion:**

Higher-ranked and larger programs exhibit a more robust Instagram presence with greater emphasis on engagement opportunities, despite these posts comprising only a small fraction of overall content. These findings highlight the potential for optimized social media strategies to enhance digital outreach and improve residency recruitment in an evolving virtual landscape.

## Introduction

Social media platforms, particularly Instagram, have transformed residency recruitment by providing integrated plastic surgery programs with innovative avenues to enhance their visibility and directly connect with prospective applicants. 1 2 Unlike traditional recruitment methods, Instagram offers a dynamic visual platform for programs to highlight their culture, showcase achievements, and provide a glimpse into daily life that resonates with applicants. 3 The COVID-19 pandemic accelerated this shift, as restrictions on in-person interactions necessitated more creative digital outreach strategies. 4

Beyond serving as an informational resource, Instagram has emerged as a vital tool for engagement. Programs are now leveraging the platform to advertise opportunities for direct applicant interaction, such as meet-and-greets, question-and-answer (Q&A) sessions, and open houses, which allow prospective candidates to gauge the personal fit and institutional environment. 5 6 In a survey of residency applicants, 97% identified personal fit within a program as a critical factor influencing their residency selection. This highlights the importance of giving applicants the chance to interact with residents and faculty and assess alignment with their personal and professional goals. 7 However, there are currently little data about how programs strategically use Instagram posts to maximize engagement and attract candidates.

This study aims to analyze Instagram activity across integrated plastic surgery residency programs, specifically examining the association between program reputation and the frequency as well as the nature of posts that promote direct engagement with prospective applicants. We hypothesize that programs with higher Doximity reputation rankings may adopt more active Instagram strategies compared to lower-ranked programs. Understanding these patterns could provide valuable insights into how programs can use social media to maintain or enhance competitiveness. This study also serves as an indirect evaluation of Instagram's role as a recruitment and engagement tool, particularly in the context of how integrated plastic surgery programs strategically deploy content to attract prospective applicants.

## Methods

Integrated plastic surgery residency programs accredited by the Accreditation Council for Graduate Medical Education for the academic year 2023 to 2024 were included in the study. The Instagram profile of each program was identified through its respective program website. All program-specific Instagram profile posts from January 1, 2020, to October 26, 2024, were examined by four independent reviewers. The total number of posts per program and posts that advertised opportunities for prospective applicants to directly interact with programs, described as “engagement posts,” were abstracted. Engagement posts were categorized based on specific content: meet-and-greets, Q&A sessions, open houses, conference meet-ups, and workshops. Advertisements for prerecorded events and subinternships were excluded because they do not provide synchronous, interactive opportunities for applicants to engage directly with program faculty or residents, which was a key criterion in defining engagement posts for this study. Primary outcomes were the percentage of engagement posts and the average annual posts per program. Programs were subsequently categorized into cohorts based on “reputation” rankings by the 2024 Doximity Residency Navigator, with groupings as “Top 20,” “21–40,” “41–60,” and “61–88.” While Doximity rankings are widely used, they are a subjective measure that may not fully capture program quality or applicant priorities.

Additionally, programs were stratified by geographic region (Midwest, Northeast, South, and West) and program size, as reported by Doximity. Program size cohorts were determined by percentile distribution of residency positions and classified as small (6–12 residents), medium (13–23 residents), and large (24–30 residents). One-way analysis of variance (ANOVA) was used to compare mean annual post counts and engagement post rates. A multivariable linear regression model was constructed to evaluate the independent associations of reputation ranking, geographic region, and program size on the average annual post count. All statistical analyses were conducted utilizing Stata 18.5 (Stata Corp LLC, College Station, TX) with statistical significance defined as a *p* -value of less than 0.05.

## Results

A total of 88 accredited integrated plastic surgery residency programs were identified. All programs had active Instagram profiles. At the start of 2020, 76/88 (86.3%) of programs had active profiles. An additional six programs had active profiles as of 2021, three as of 2022, two as of 2023, and one as of 2024. A total of 13,719 posts were shared since 2020, with an average of 32.4 annual posts per program. Of these posts, 369 (2.7%) were engagement posts, composed of meet-and-greets (71.3%), Q&A sessions (13.8%), open houses (10.6%), workshops (2.4%), and research conference meet-ups (1.9%; Table 1 ).

**Table 1:** Total posts and engagement posts of all programs

	Number of posts
Total posts	13,719
Total engagement posts	369 (2.7%)
Meet-and-greets	263 (71.3%)
Q&A sessions	51 (13.8%)
Open houses	39 (10.6%)
Workshops	9 (2.4%)
Research conference meet-ups	7 (1.9%)

Abbreviation: Q&A, question and answer.

**Fig. 1:**
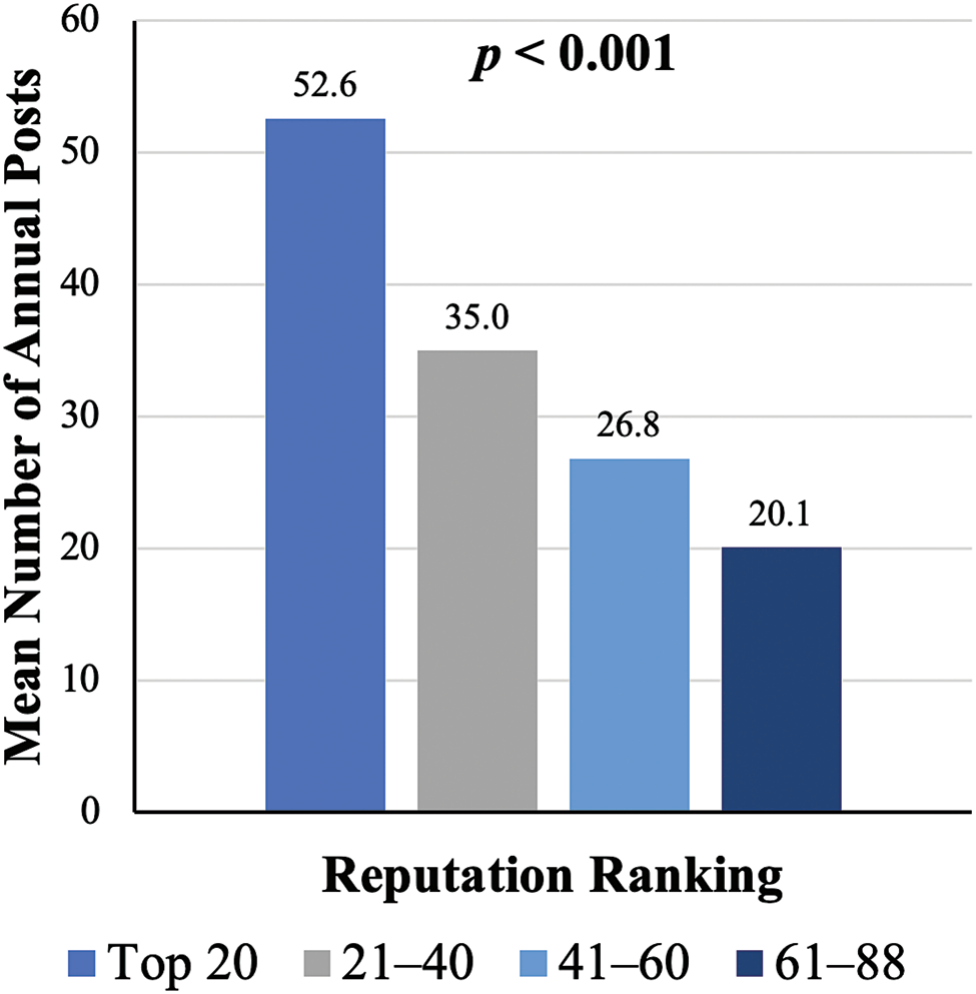
Mean number of annual Instagram posts per program by rank cohort. Bar heights represent the average number of posts per year for programs in each reputation-based cohort. The overall *p* -value reflects the statistical significance of differences in mean post frequency across the four ranking groups. Bold values represent a significant *p* -value <0.05.

**Fig. 2:**
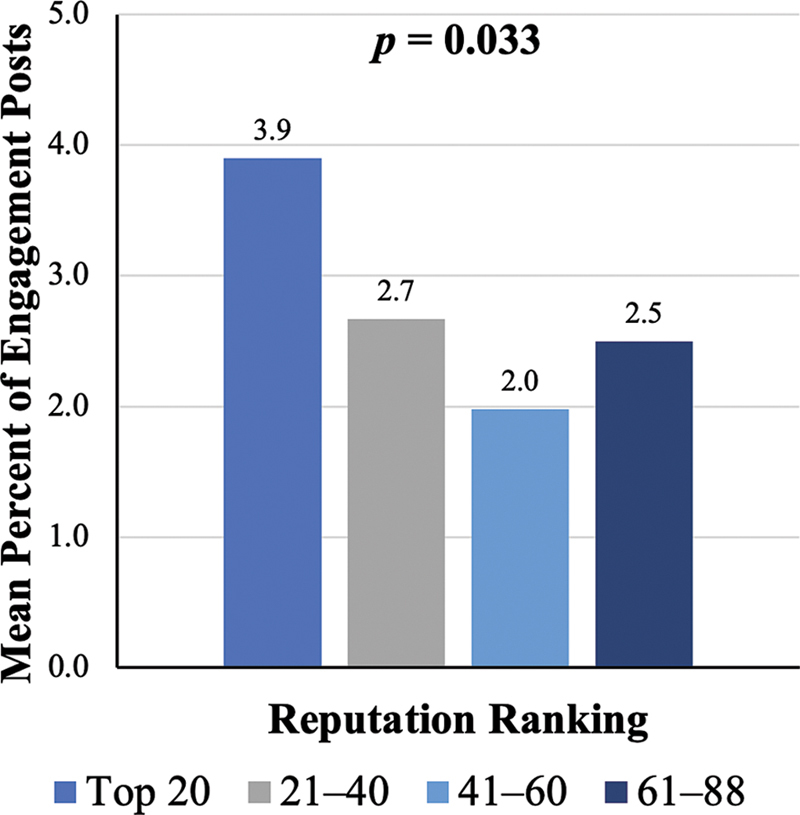
Mean percentage of engagement posts per program by rank cohort. Bars show the average proportion of posts classified as engagement posts in each reputation cohort. The overall *p* -value indicates whether there were significant differences in engagement post rates among the ranking groups. Bold values represent a significant *p* -value <0.05.

Across reputation ranking cohorts, mean program size decreased significantly with values of 20.7 ± 4.95 for the Top 20, 16.35 ± 5.10 for 21–40, 12.6 ± 4.73 for 41–60, and 10.5 ± 3.51 for 61–88 ( *p* < 0.001; Table 2 ). Geographic distribution was similar across these groups. The mean number of annual posts was highest in the Top 20 programs at 52.6, followed by 35.0 for 21–40, 26.8 for 41–60, and 20.1 for 61–88 ( *p* < 0.001; Fig. 1 ). The mean proportion of engagement posts was 3.9% for the Top 20, 2.7% for 21–40, 2.0% for 41–60, and 2.5% for 61–88 ( *p* = 0.033; Fig. 2 ).

**Table 2:** Demographics and post frequency by reputation ranking

	Reputation ranking				*p* -Value
	Top 20	21–40	41–60	60–88	
Program size, mean ± SD	20.7 ± 5.0	16.4 ± 5.1	12.6 ± 4.7	10.5 ± 3.5	**<0.001**
West (%)	6 (30%)	3 (15%)	4 (20%)	2 (7%)	0.21
South (%)	4 (20%)	7 (35%)	4 (20%)	11 (39%)	0.35
Midwest (%)	5 (25%)	6 (30%)	5 (25%)	7 (25%)	0.98
Northeast (%)	5 (25%)	4 (20%)	7 (35%)	8 (29%)	0.75
Engagement post rate, mean ± SD	3.9% ± 2.9%	2.7% ± 1.5%	2.0% ± 2.0%	2.5% ± 1.9%	**0.033**
Posts per year, mean ± SD	52.6 ± 32.2	35.0 ± 21.8	26.8 ± 15.8	20.1 ± 9.8	**<0.001**

Abbreviation: SD, standard deviation.

Bold values represent a significant *p* -value <0.05.

When examining differences by geographic region, the mean program size and reputation ranking did not differ significantly across regions ( Table 3 ). However, the number of posts per year varied significantly, with means of 48.80 in the West, 35.57 in the Midwest, 27.15 in the South, and 24.75 in the Northeast ( *p* = 0.008), while the engagement post rate was similar across regions ( *p* = 0.81).

**Table 3:** Demographics and post frequency by geographic region

	Geographic region				*p* -Value
	West	Midwest	South	Northeast	
Program size, mean ± SD	15.6 ± 6.3	15.0 ± 6.2	13.8 ± 5.8	14.5 ± 5.8	0.82
Rep rank, mean ± SD	36.2 ± 25.0	44.6 ± 25.5	48.9 ± 25.1	44.8 ± 26.7	0.51
Engagement post rate, mean ± SD	2.8% ± 1.8%	2.8% ± 2.2%	3.0% ± 1.6%	2.4% ± 2.8%	0.81
Posts per year, mean ± SD	48.8 ± 38.0	35.6 ± 25.1	27.2 ± 13.6	24.8 ± 13.3	**0.008**

Abbreviation: SD, standard deviation.

Bold values represent a significant *p* -value <0.05.

Further analysis by program size showed that large programs ( *n* = 14) had a mean reputation ranking of 16 ± 14.44, medium programs ( *n* = 23) had 32.48 ± 22.05, and small programs ( *n* = 51) had 57.75 ± 19.87 ( *p* < 0.001; Table 4 ). Although regional representation did not differ significantly by program size, large programs posted more frequently, averaging 56.86 ± 37.12 posts per year, compared to 36.87 ± 21.27 for medium programs and 23.65 ± 13.06 for small programs ( *p* < 0.001). The engagement post rate did not differ significantly across program sizes ( *p* = 0.11).

**Table 4:** Demographics and post frequency by program size

	Program size			*p* -Value
	Large	Medium	Small	
*N*	14	23	51	
Rep rank, mean ± SD	16.0 ± 14.4	32.5 ± 22.1	57.7 ± 19.9	**<0.001**
West (%)	2 (14%)	6 (26%)	7 (14%)	0.41
South (%)	3 (21%)	5 (22%)	18 (35%)	0.38
Midwest (%)	5 (36%)	6 (26%)	12 (24%)	0.66
Northeast (%)	4 (29%)	6 (26%)	14 (28%)	0.99
Engagement post rate, mean ± SD	3.6% ± 3.3%	3.1% ± 2.1%	2.3% ± 1.8%	0.11
Posts per year, mean ± SD	56.9 ± 37.1	36.9 ± 21.3	23.6 ± 13.1	**<0.001**

Abbreviation: SD, standard deviation.

Multivariable regression analysis revealed that the Top 20 programs posted 17.03 more times per year than programs ranked 61–88 (95% CI: 1.96, 32.10; *p* = 0.027), while differences for programs ranked 21–40 (B = 7.16; *p* = 0.265) and 41–60 (B = 3.21; *p* = 0.580) did not reach significance ( Table 5 ). Among geographic regions, only the West showed a significant increase in posts per year compared to the Northeast (B = 20.76; 95% CI: 7.87, 33.64; *p* = 0.002). Regarding program size, large programs posted 22.11 more times per year than small programs (95% CI: 7.24, 36.98; *p* = 0.004), while medium programs did not reach significance compared to small programs (B = 4.05; *p* = 0.488). The model was significant ( *F* [8, 79] = 6.80, *p* < 0.001; adjusted *R* ^2^ = 0.35), with no evidence of multicollinearity or heteroscedasticity.

**Table 5:** Multivariable linear regression on annual Instagram post rate

Variable	Category	Coefficient	Standard error	*p* -Value	95% CI
Rank	60–88	Reference	–	–	–
	Top 20	17.0299	7.5727	**0.027**	1.9568–32.1030
	21–40	7.1602	6.3783	0.265	−5.5355–19.8560
	41–60	3.2125	5.7881	0.58	−8.3084–14.7334
Region	Northeast	Reference	–	–	–
	Midwest	9.0594	5.629	0.112	−2.1449–20.2636
	South	4.4075	5.5262	0.428	−6.5920–15.4071
	West	20.7579	6.4733	**0.002**	7.8732–33.6427
Size	Small	Reference	–	–	–
	Medium	4.045	5.8061	0.488	−7.5117–15.6018
	Large	22.1139	7.4705	**0.004**	7.2442–36.9836

Bold values represent a significant *p* -value < 0.05.

## Discussion

Our analysis revealed that integrated plastic surgery programs with higher Doximity reputation rankings not only maintain a more active Instagram presence but also provide more opportunities for direct applicant engagement, particularly through meet-and-greets. Top-ranked and larger programs posted significantly more often than their lower-ranked and smaller counterparts, suggesting that a robust social media strategy may reinforce a program's reputation and help attract top applicants.

Social media now plays a central role in how applicants learn about residency programs and stay informed about opportunities. In recent years, integrated plastic surgery programs have expanded their presence on platforms like Instagram, Facebook, and Twitter. 1 8 Instagram, in particular, has emerged as a key resource for applicants to explore program culture and resident life, ultimately aiding their ranking decisions. Applicants often view resident involvement in social media as a reflection of institutional culture, with programs that encourage resident-authored posts signaling an autonomous and supportive environment. 9 Furthermore, research has shown that applicants value perceptions of resident happiness as a key consideration when ranking programs, while concerns about a program being “malignant” have a notably negative impact. 7 Consistent social media content may thus shape program perceptions for future candidates, particularly when facilitating direct outreach.

In our study, engagement-focused posts represented only a small fraction of Instagram content from integrated plastic surgery programs, ranging from 2.5% to 3.9% across cohorts. These posts offer applicants an opportunity to gain deeper insights into a program, foster meaningful interactions, and assess personal fit. Comparative studies across specialties, including family medicine and otolaryngology, have reported similar patterns, where an active Instagram presence correlates with increased applicant engagement and higher perceived program quality. 10 11 Recommendations from prior research, such as the “Twelve Tips for Utilizing Residency Program Social Media Accounts,” suggest that incorporating interactive sessions can further strengthen a program's digital appeal. 12 With away rotations limited by cost and logistics, social media offers an accessible, low-cost way for applicants to explore programs more personally than static websites allow. 13 14 The overall scarcity of engagement posts suggests an important area for improvement. A 2022 study analyzing 40 plastic surgery residency programs found that engagement rates peaked in May and June, with virtual meet-and-greets held over Zoom being the most common activity. 15 Therefore, programs can potentially enhance outreach by optimizing the timing and frequency of these posts during pivotal moments in the application cycle, such as when applications open, when interview invitations are extended, and as ranking deadlines approach. 16

The next step is to explore qualitative and longitudinal approaches. Future studies could use focus groups or in-depth interviews to better understand how different types of social media content influence applicant perceptions and decision-making processes. Longitudinal methodologies, such as serial surveys or matched cohort tracking of applicant behavior over multiple application cycles, would offer insight into the sustained impact of digital engagement. Comparing the effectiveness of different platforms, such as Instagram Stories versus permanent posts, could reveal which features most effectively convey a program's identity and culture. These designs are feasible to implement through coordinated efforts among programs or national societies, particularly during the standardized residency recruitment timeline. Ultimately, developing best practice guidelines based on these findings and those from similar studies will be instrumental in helping programs standardize their digital outreach efforts while maintaining authenticity and diversity in their messaging.

Our additional analyses based on program size and geographic region provide further insights into Instagram dynamics. Larger programs posted more frequently on average (56.9 posts per year for large programs vs. 23.7 for small programs), and while the difference in engagement post rate across program sizes was not statistically significant, there was a slight trend toward a higher percentage among larger programs. Similarly, programs in the West posted more frequently than those in the Northeast, although engagement post rates were comparable. Furthermore, multivariable regression analysis confirmed that reputation ranking, program size, and regional context are independently associated with posting frequency. After controlling for these factors, the Top 20 programs posted, on average, 17 more times per year than programs ranked 61–88, and large programs posted about 22 more times per year than small programs. Programs in the West also posted roughly 21 more times per year compared with those in the Northeast. These regional differences may reflect underlying institutional or geographic variation in digital communication priorities. Programs in the Western United States may have greater access to digital marketing support, more emphasis on social media engagement from departmental leadership, or a culture that encourages online presence and transparency. Additionally, regional trends in technology adoption and applicant expectations could drive more consistent online outreach in some areas compared to others. These results indicate that reputation, program size, and regional context may all be related to differences in posting frequency, suggesting that multiple factors could be associated with an active Instagram presence.

The practical significance of these differences lies in how prospective applicants interpret and interact with program content. A more active Instagram presence may enhance a program's visibility during critical stages of the recruitment cycle, shaping perceptions of transparency, responsiveness, and overall organizational culture. Higher frequencies of engagement posts may signal to applicants that a program is approachable and invested in applicant engagement, potentially influencing decisions to apply, accept interview invitations, or rank programs more highly. Although the absolute difference in engagement post rates appears modest, such variation may still meaningfully shape applicant impressions over time, particularly when engagement content is concentrated around key decision points in the recruitment cycle. Residency programs across reputation tiers may benefit from leveraging social media more effectively by increasing the frequency and relevance of their posts. As appreciated by Irwin et al, expanding social media engagement strategies may help programs connect with a diverse applicant pool and enhance overall visibility. 3

However, the differences in posting frequency may be related not only to reputation or region, but also to underlying factors such as resource allocation and institutional priorities. Top-ranked and larger programs may be more likely to have dedicated digital marketing teams or allocated budgets that enable them to produce high-quality, real-time posts, thereby enhancing their overall online engagement. A program's digital literacy, reflected in the willingness of its faculty and residents to adopt innovative communication tools, would further amplify social media efficacy, creating a reinforcing cycle in which an active and authentic online presence boosts reputation and, in turn, attracts a more competitive applicant pool.

Compared to previous descriptive studies, our use of a 4-year dataset and multivariable linear regression allows for a more robust analysis of independent predictors of Instagram activity. The cohort-based stratification by reputation, region, and size further enables comparative insights that extend beyond surface-level observations. These methodological strengths offer a more comprehensive understanding of how institutional characteristics shape digital engagement patterns.

This study has several limitations that should be acknowledged. First, our analysis focused solely on Instagram, which has emerged as the most widely used and content-rich platform for both plastic surgery residency programs and prospective applicants. Other platforms, such as Twitter, YouTube, and Facebook, were excluded to maintain analytic consistency, although we recognize that cross-platform strategies may influence applicant perceptions and represent a valuable avenue for future study. 3 17 However, a broader evaluation of social media activity could provide a more comprehensive understanding of outreach strategies. Furthermore, fleeting content such as Instagram Live and Stories was not analyzed in this study, as these features are only temporarily available, despite serving as avenues for real-time interaction. Their exclusion may lead to an underestimation of total applicant engagement volume. Also, we relied on Doximity rankings as one measure of residency program reputation, which provides a limited perspective. Doximity's Residency Navigator employs a multifactorial methodology that integrates resident satisfaction surveys, research data, and objective metrics. 18 While useful, the subjective nature of reputation assessment and variability in resident self-reported data suggest that these rankings should be interpreted with caution. Additionally, we did not assess engagement quality metrics such as likes, comments, or reach, which may provide more nuanced insights into how posts are received by applicants. Our analysis also lacks direct applicant input, so we cannot determine how different types of posts influence applicant behavior or decision-making. Finally, although posts were independently reviewed, some degree of subjectivity in classifying “engagement posts” may introduce classification bias.

The role of virtual platforms in residency recruitment will continue to evolve in the coming years. Identifying the most effective types of online content able to support candidates during the application process is a key area for further study. Future directions include administration of national surveys to quantify the impact of Instagram engagement on ultimate interviewing and ranking decisions, as well as more detailed analyses that explore how program region and size shape social media strategies.

### Conclusion

This study reveals key differences in patterns of Instagram activity among integrated plastic surgery programs. Higher-ranked and larger programs tended to post more frequently, and regional differences were observed in overall posting frequency. While we did not assess the direct impact on applicant recruitment, these variations in digital engagement may affect program visibility and perceptions of institutional culture. These findings suggest a pathway for residency programs to consider optimizing their online presence and underscore the need for future research to evaluate how social media impacts recruitment outcomes in a rapidly evolving digital landscape.
